# Protein expression patterns of cell cycle regulators in operable breast cancer

**DOI:** 10.1371/journal.pone.0180489

**Published:** 2017-08-10

**Authors:** Flora Zagouri, Vassiliki Kotoula, George Kouvatseas, Maria Sotiropoulou, Triantafyllia Koletsa, Theofani Gavressea, Christos Valavanis, Helen Trihia, Mattheos Bobos, Georgios Lazaridis, Angelos Koutras, George Pentheroudakis, Pantelis Skarlos, Dimitrios Bafaloukos, Niki Arnogiannaki, Sofia Chrisafi, Christos Christodoulou, Pavlos Papakostas, Gerasimos Aravantinos, Paris Kosmidis, Charisios Karanikiotis, George Zografos, Christos Papadimitriou, George Fountzilas

**Affiliations:** 1 Department of Clinical Therapeutics, Alexandra Hospital, National and Kapodistrian University of Athens School of Medicine, Athens, Greece; 2 Department of Pathology, Aristotle University of Thessaloniki, School of Health Sciences, Faculty of Medicine, Thessaloniki, Greece; 3 Laboratory of Molecular Oncology, Hellenic Foundation for Cancer Research/Aristotle University of Thessaloniki, Thessaloniki, Greece; 4 Health Data Specialists Ltd, Athens, Greece; 5 Department of Pathology, Alexandra Hospital, Athens, Greece; 6 Department of Pathology, Iaso Women’s Hospital, Athens, Greece; 7 Department of Pathology, Metaxas Cancer Hospital, Piraeus, Greece; 8 Department of Medical Oncology, Papageorgiou Hospital, Aristotle University of Thessaloniki, School of Health Sciences, Faculty of Medicine, Thessaloniki, Greece; 9 Division of Oncology, Department of Medicine, University Hospital, University of Patras Medical School, Patras, Greece; 10 Department of Medical Oncology, Ioannina University Hospital, Ioannina, Greece; 11 Department of Radiotherapy, Metropolitan Hospital, Piraeus, Greece; 12 First Department of Medical Oncology, Metropolitan Hospital, Piraeus, Greece; 13 Department of Surgical Pathology, Agios Savas Anticancer Hospital, Athens, Greece; 14 Second Department of Medical Oncology, Metropolitan Hospital, Piraeus, Greece; 15 Oncology Unit, Hippokration Hospital, Athens, Greece; 16 Second Department of Medical Oncology, Agii Anargiri Cancer Hospital, Athens, Greece; 17 Second Department of Medical Oncology, Hygeia Hospital, Athens, Greece; 18 Department of Medical Oncology, 424 Army General Hospital, Thessaloniki, Greece; 19 Breast Unit, National and Kapodistrian University of Athens School of Medicine, Athens, Greece; 20 Aristotle University of Thessaloniki, Thessaloniki, Greece; University of South Alabama Mitchell Cancer Institute, UNITED STATES

## Abstract

**Background-Aim:**

To evaluate the prognostic role of elaborate molecular clusters encompassing cyclin D1, cyclin E1, p21, p27 and p53 in the context of various breast cancer subtypes.

**Methods:**

Cyclin E1, cyclin D1, p53, p21 and p27 were evaluated with immunohistochemistry in 1077 formalin-fixed paraffin-embedded tissues from breast cancer patients who had been treated within clinical trials. Jaccard distances were computed for the markers and the resulted matrix was used for conducting unsupervised hierarchical clustering, in order to identify distinct groups correlating with prognosis.

**Results:**

Luminal B and triple-negative (TNBC) tumors presented with the highest and lowest levels of cyclin D1 expression, respectively. By contrast, TNBC frequently expressed Cyclin E1, whereas ER-positive tumors did not. Absence of Cyclin D1 predicted for worse OS, while absence of Cyclin E1 for poorer DFS. The expression patterns of all examined proteins yielded 3 distinct clusters; (1) Cyclin D1 and/or E1 positive with moderate p21 expression; (2) Cyclin D1 and/or E1, and p27 positive, p53 protein negative; and, (3) Cyclin D1 or E1 positive, p53 positive, p21 and p27 negative or moderately positive. The 5-year DFS rates for clusters 1, 2 and 3 were 70.0%, 79.1%, 67.4% and OS 88.4%, 90.4%, 78.9%, respectively.

**Conclusions:**

It seems that the expression of cell cycle regulators in the absence of p53 protein is associated with favorable prognosis in operable breast cancer.

## Introduction

The significance of cell cycle mediators in breast carcinogenesis is currently well established. Specifically, deregulation of crucial genes that control cell cycle checkpoints has been noted in various breast carcinomas [1]. Moreover, dysfunction or loss of these genes can also mediate resistance to chemotherapeutic agents.

Cyclin D1 and its associated cyclin-dependent kinases (CDK4 and CDK6) are central mediators in the transition from G1 to S phase [2]. In primary breast cancer, it has been shown that the gene encoding cyclin D1 is amplified in 15% of the cases and overexpressed in 30–50%[3]. Of note, elevated levels of cyclin D1 protein have been associated with poor prognosis, whilst overexpression of cyclin D1 has been more commonly found in hormone receptor (HR) positive breast cancer cases [3]. Interestingly enough, the activity of CDK4 has been found not to strictly follow cyclin D1 expression in breast cancer cell lines, a finding suggesting that CDK4-independent functions of cyclin D1 may contribute to its biological effects as an oncogene in breast cancer [4, 5].

The maximum levels of cyclin E are correlated with a peak in the enzymatic function of the cyclin E-CDK2 complex, which is important in the transition from G1 to S phase [6]. Oncogenic effects of cyclin E deregulation, especially overexpression of shortened or low molecular weight forms of this protein, are reinforced by loss of regulatory control through p53 to promote tumor progression. Expression of cyclin E protein promotes progression into phase S, an activity opposed by p53-regulated activation of checkpoint controls or apoptosis. Loss of p53 function is an escape hatch by which tumor cells can avoid cell cycle arrest or cell death and progress through further stages of unchecked deregulation and growth [7].

Towards this direction, loss of function-expression of p21 (CDKN1A) and p27 (CDKN1B), the two G1-checkpoint CDK inhibitors, has been implicated in breast carcinogenesis and progression of the disease [8]. Moreover, accumulating data suggest that functional loss of p21 or p27 can mediate a drug-resistance phenotype.

Of note, there are aspects in this protein network, in various breast cancer subtypes, that have not been fully understood. New data on the field are more than warranted taking into consideration the introduction of the second generation of highly specific cyclin D1/CDK4/CDK6 inhibitors, agents highly active in metastatic breast cancer [9]. According to our knowledge, this is the first study trying to evaluate the prognostic role of elaborate molecular clusters encompassing cyclin D1, cyclin E1, p21 (CDKN1A), p27 (CDKN1B) and p53 in the context of various breast cancer subtypes.

## Materials and methods

### Study population

The study was performed on formalin-fixed paraffin-embedded (FFPE) tissues from a series of tumors derived from patients with operable intermediate/high-risk early breast cancer who had been treated within the frame of two randomized phase III trials by the Hellenic Cooperative Oncology Group (HeCOG). The HE10/97 trial [10] was a randomized phase III trial in patients with high-risk node-negative or intermediate/high-risk node-positive operable breast cancer, comparing four cycles of epirubicin (E) followed by four cycles of intensified CMF (E-CMF) with three cycles of E, followed by three cycles of paclitaxel (T, Taxol, Bristol Myers-Squibb, Princeton, NJ) followed by three cycles of intensified CMF (E-T-CMF). All cycles were given every two weeks with G-CSF support. Dose intensity of all drugs in both treatment arms was identical, but cumulative doses and duration of chemotherapy period differed. In total, 595 eligible patients entered the study in a period of 3.5 years (1997–2000).

The HE10/00 trial [11, 12] was a randomized phase III trial, in which patients were treated with E-T-CMF (exactly as in the HE10/97 trial) or with four cycles of epirubicin/paclitaxel (ET) combination (given on the same day) every three weeks followed by three cycles of intensified CMF every two weeks (ET-CMF). By study design, the cumulative doses and the chemotherapy duration were identical in the two arms but dose intensity of epirubicin and paclitaxel was double in the E-T-CMF arm. A total of 1,086 eligible patients with node-positive operable breast cancer were accrued in a period of 5 years (2000–2005).

Treatment schedules for the two studies are shown in S1 Table. All patients had undergone modified radical mastectomy (MRM) or breast-conserving surgery (BCS). Patients with HER2-positive tumors had received trastuzumab upon relapse. Clinical protocols were approved by local regulatory authorities and were also included in the Australian New Zealand Clinical Trials Registry (ANZCTR) and allocated the following Registration Numbers: ACTRN12611000506998 (HE10/97) and ACTRN12609001036202 (HE10/00). The present translational research protocol was approved by the Bioethics Committee of the Aristotle University of Thessaloniki School of Medicine under the general title ‘‘Molecular investigation of the predictive and/or prognostic role of important signal transduction pathways in breast cancer” (A7150/18-3-2008). All patients signed a study-specific written informed consent before randomization, which in addition to giving consent for the trial allowed the use of their biological material for future research purposes. Of note, tissues studied were taken before chemotherapy administration.

#### Immunohistochemistry and FISH assays for molecular subtyping of breast carcinomas

Immunohistochemistry (IHC) for ER, PgR, HER2, Ki67, CK5 and EGFR proteins and triple FISH for HER2/TOP2A/CEN17 DNA probes data used in this study have previously been described [13]. Briefly, HER2 was scored with IHC in a scale from 0–3, with intense membrane staining in >30% invasive tumor cells classified as positive (3+ staining) [14]. Cut-offs were set for ER and PgR at ≥ 1% positivity in tumor cell nuclei [15]. For Ki67 labeling, 14% was used as cut-off to categorize low (<14%) and high (≥14%) protein expression for distinguishing Luminal A and B tumors [16]. Ki67 was evaluated as a continuous variable (% of positively stained tumor cell nuclei) and the highest score for each tissue microarray tumor core from the same case was recorded. FISH was evaluated in twenty tumor cell nuclei [17]. The HER2 gene was classified as amplified for HER2/CEN17 ratios ≥2.2 [14], or for mean HER2 copy numbers >6 [18]. Cytokeratin 5 (CK5) and epidermal growth factor receptor (EGFR) immunostains were applied for typing basal-like carcinomas. The cut-off was set at ≥1% for EGFR positivity [19].

#### Immunohistochemistry for p21, p27, p53, p63, cyclin D1, cyclin E1 and CD117

IHC was performed using the Bond Max and Bond III autostainers (Leica Microsystems, Wezlar, Germany). For p21 protein expression, the mouse monoclonal antibody (Clone SX118, Dako, DK; 1:60 dilution, 30' incubation) was used. Proteins p27 and p63 were assessed using mouse monoclonal antibodies (clone SX53G8, Dako; at dilution 1:150, 30' incubation and clone 7JUL at 1:50 dilution, 1h incubation at room temperature respectively). p53 protein expression was assessed with anti-p53 clone DO7 (DAKO) at 1:100 dilution, upon antigen retrieval in citric acid for 20 min. Cyclin D1 protein expression was assessed using the rabbit monoclonal antibody (clone SP4, Spring Bioscience, USA; 1:200 dilution, 1h incubation at room temperature), cyclin E1 protein using the rabbit monoclonal antibody (clone EP435E, Novus Biologicals, Littleton, CO; 1:80 dilution, 30' incubation) and CD117 with the polyclonal rabbit antibody (code A4502, Dako). The antigen–antibody complex was visualized using DAB as a chromogen. Slides were counterstained with Mayer’s hematoxylin for 10 min (Leica), washed in water, dehydrated and mounted.

#### Interpretation of the p21, p27, p53, p63, cyclin D1, cyclin E1 and CD117 immunostains

**Fig 1:**
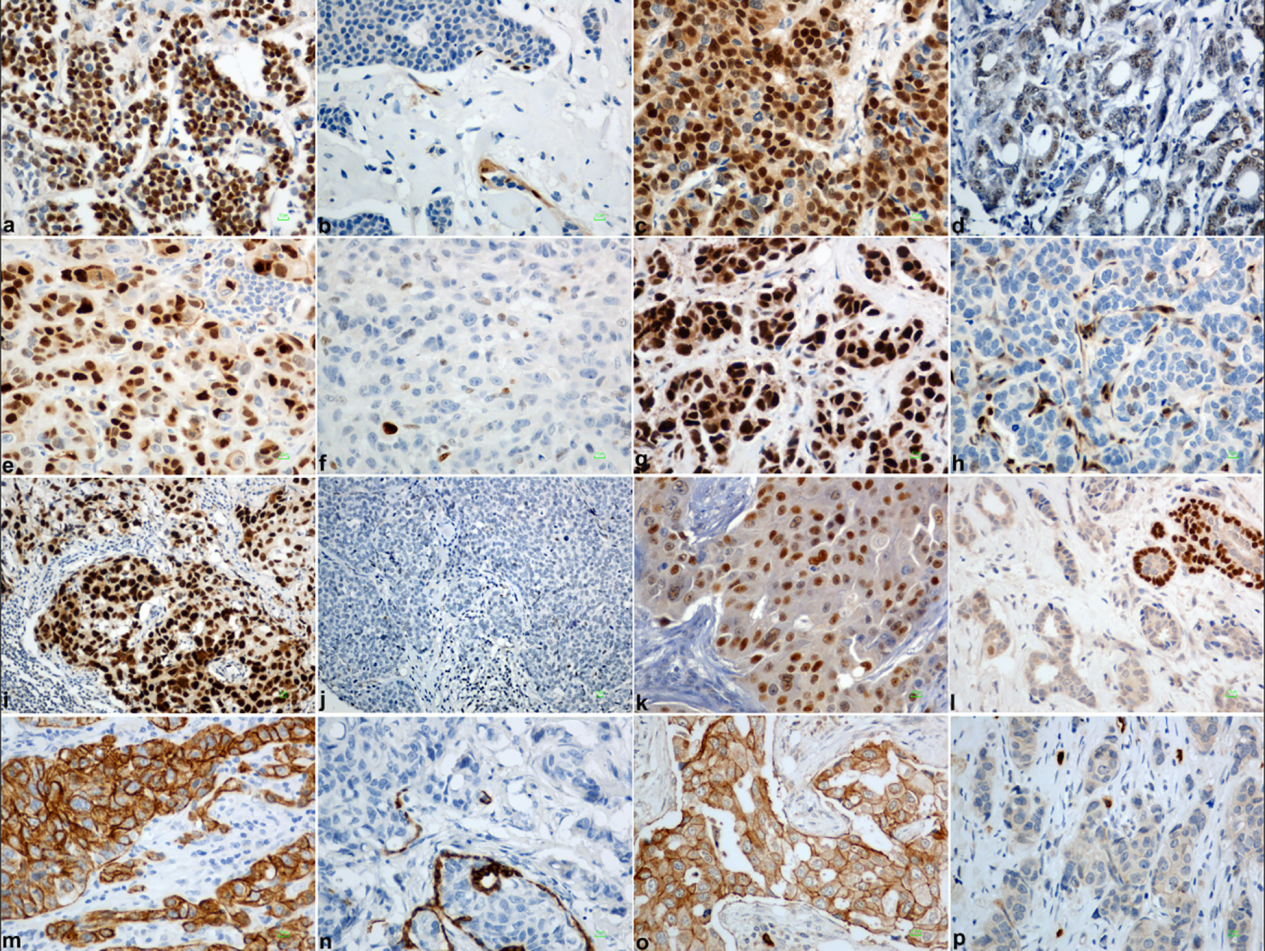
Representative images of invasive breast carcinomas (IBC) stained for Cyclin D1 (a, b), Cyclin E1 (c, d), p21 (e, f), p27 (g, h), p53 (i, j), p63 (k, l), CK5/6 (m, n) and CD117 (o, p). The panels a, c, e, g, i, k, m, o represent positive cases for each protein, whereas the panels b, d, f, h, i, j, l, and p, tumors with absence or low expression of the corresponding protein. Bar 10μm.

p21 and p27 nuclear immunostaining were classified as 3 scale variables whereby ≤10% corresponded to low or negative; 11–50% to moderate; and, >50% to high expression (slightly modified from [20, 21]). For p63 the cut-off was set at ≥5% of tumor cells with nuclear expression of any intensity [22], for cyclin E1 at >50% of tumor cells with nuclear staining [23], while for CD117, any membranous or cytoplasmic staining in tumor cells was considered as positive [24]. In order to avoid missing cut-offs with prognostic utility for patient outcome, we carried out receiver-operator curve (ROC) analysis for cyclin D1, CK5 and p53 proteins, as described in detail in the statistical methods section. Characteristic examples for the performance of staining and for various levels of protein expression for these targets is shown in Fig 1.

#### Statistical methods

Frequencies with corresponding percentages were used for displaying categorical data, while various measures (mean, standard deviation, range) were used regarding continuous. The chi-square test was performed in order to investigate for possible associations between the examined markers and clinical, tumor or other characteristics.

**Fig 2:**
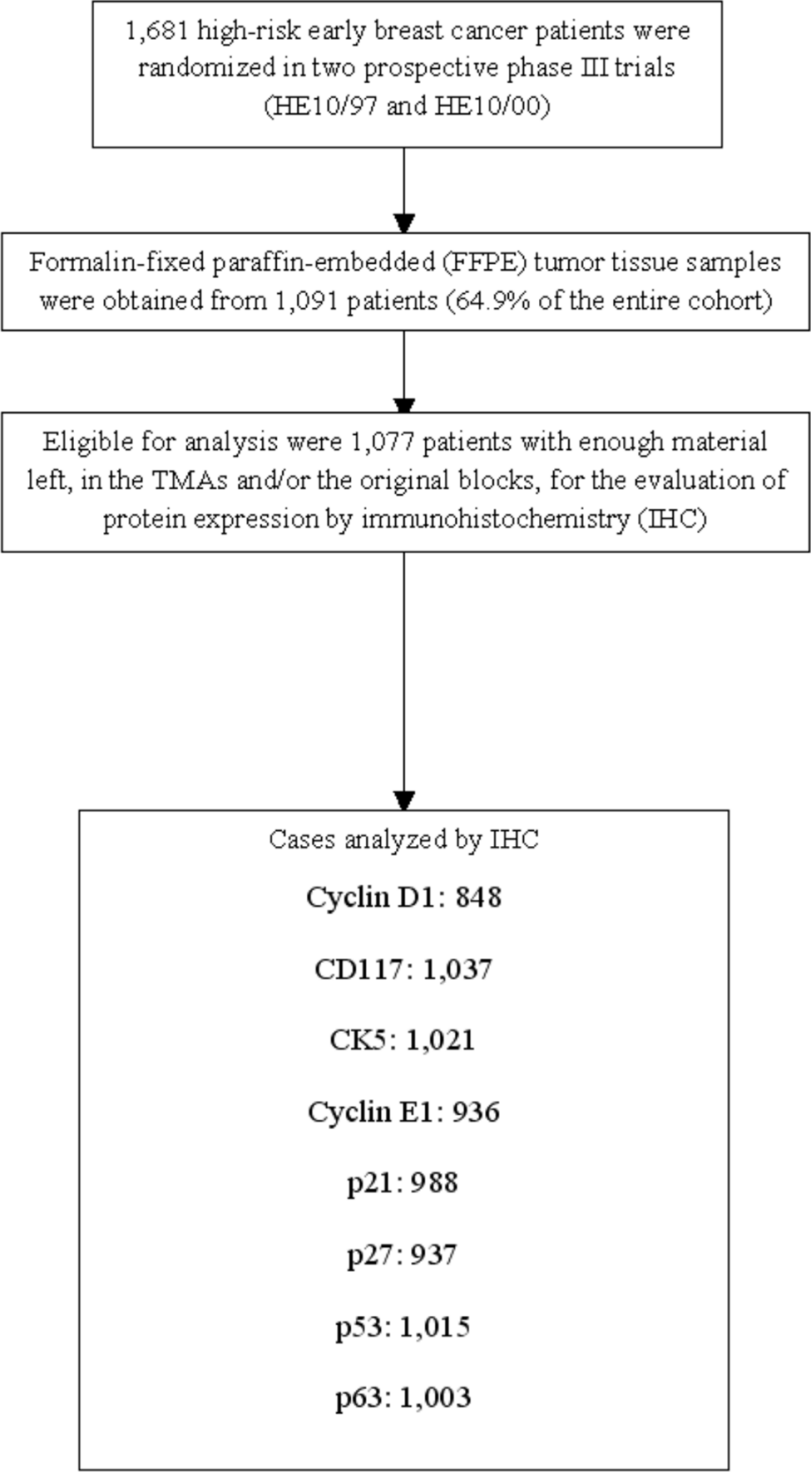
REMARK diagram.

Disease-free survival (DFS) was measured from the date of diagnosis until verified disease progression, death from any cause or date of last contact, whichever occurred first, while overall survival (OS) from diagnosis until death or last contact. The product limit method and Kaplan-Meier curves were used for estimating and plotting time-to-event distributions, while log-rank tests were used for assessing statistically significant differences. Concerning three markers, CK5, p53 and cyclin D1, the optimal cut-off points for separating high and low expression were calculated by ROC curve analysis with DFS at five years as the outcome variable. The cut offs obtained were 12.5% for CK5, 72.5% for p53 and 15% for cyclin D1. The REMARK diagram [25] for the study is shown in Fig 2.

Jaccard distances were computed for the markers and the resulted matrix was used for conducting unsupervised hierarchical clustering (using the Ward’s minimum variance method), in order to identify distinct groups correlating with prognosis.

Multivariate Cox regression analysis was performed, with a backward selection procedure in order to identify significant factors. The clinical variables that were assessed in multivariate analysis were: surgery (modified radical vs. partial/simple mastectomy), ER/PgR positivity, tumor size (≤2 vs. >2 cm), number of positive nodes (0–3 vs. ≥4), treatment (HE1000-A: E-T-CMF vs. HE1000-B: ET-CMF vs. HE1097-A: E-T-CMF vs. HE1097-B: E-CMF), subtypes (Luminal A vs. Luminal B vs. Luminal HER2 vs. HER2-Enriched vs. TNBC), histological grade (1–2 vs. 3–4), continuous Ki67 (%) and resulted clusters. It was decided to include in the analysis the clusters as independent factors and not the immunohistochemical markers per se, since the high correlations observed between the markers would render the results highly unstable (multi-collinearity effect).

In multivariate analysis significance was determined at the level of 15% and in univariate at 5%, while all tests were two-sided. The statistical analysis complied with the reporting recommendations for tumor marker prognostic studies [25] and was performed using the SAS software (SAS for Windows, version 9.3, SAS Institute Inc., Cary, NC).

## Results

### Basic patient characteristics

Patient and tumor characteristics at baseline are presented in Table 1. In total, 1077 patients were included in this study, with median age of 53 years (range 22–79 years); 53.2% of cases were postmenopausal. The median number of cycles administered to patients was eight. Eighty percent of cases were ductal carcinomas, while luminal A and B were the most common subtypes (59.8%). At diagnosis, 68.2% of tumors were larger than 2 cm, whereas 59.6% of patients had four or more positive lymph nodes. The tumors were ER/PgR positive in 77.2% of cases. The majority of patients (68.4%) underwent modified radical mastectomy, while only 15.2% of the patients had not received taxanes.

**Table 1:** Patients’ disease characteristics andfrequencies-percentages of the examined markers—N (%).

Patients	N	1077
Age	Mean (SD)	52.8 (11.3)
	Median (Range)	53 (22–79)
Number of cycles	Mean (SD)	7.9 (1.3)
	Median (Range)	8 (0–10)
		N (%)
Menopausal status	Peri (≤2 years without menses)	22 (2)
	Post	574 (53.2)
	Pre	481 (44.6)
Histological grade	1–2	537 (49.8)
	3–4	535 (49.6)
	Not reported	5 (0.4)
Histological type	Ductal	867 (80.6)
	Inflammatory	6 (0.6)
	Lobular	111 (10.4)
	Mixed	80 (7.4)
	Other	13 (1.2)
Tumor size	≤2	342 (31.8)
	>2	735 (68.2)
Number of positive nodes	1–3	436 (40.4)
	≥4	641 (59.6)
Surgery (grouped)	Modified radical	736 (68.4)
	Partial/Simple mastectomy	339 (31.4)
	Not reported	2 (0.2)
Lymph invasion	Yes	463 (43)
	No	361 (33.6)
	Not reported	253 (23.4)
ER status	Positive	788 (73.2)
	Negative	289 (26.8)
PgR status	Positive	722 (67)
	Negative	355 (33)
ER/PgR status	Positive	832 (77.2)
	Negative	245 (22.8)
Subtypes	Luminal A	253 (23.4)
	Luminal B	392 (36.4)
	Luminal-HER2	139 (13)
	HER2-enriched	109 (10.2)
	TNBC	130 (12)
	Not reported	54 (5)
Treatment group	HE1000-A: E-T-CMF	389 (36.2)
	HE1000-B: ET-CMF	385 (35.8)
	HE1097-A: E-T-CMF	139 (13)
CCND1 (N = 848)	Negative	186 (21.9)
	Positive	662 (78.1)
CD117 (N = 1037)	Negative	985 (95.0)
	Positive	52 (5.0)
CK5 (N = 1021)	Negative	919 (90.0)
	Positive	102 (10.0)
Cyclin E1 (N = 936)	Negative	501 (53.5)
	Positive	435 (46.5)
p21 (N = 988)	≤10%	752 (76.1)
	11–50%	186 (18.8)
	>50%	50 (5.1)
p27 (N = 937)	≤10%	187 (20)
	11–50%	167 (17.8)
	>50%	583 (62.2)
p53 (N = 1015)	Negative	846 (83.3)
	Positive	169 (16.7)
p63 (N = 1003)	Negative	957 (95.4)
	Positive	46 (4.6)

#### Immunohistochemical expression of markers

Table 1 presents the results pertaining to the immunohistochemical expression of the examined markers. The majority of tumors were positive to cyclin D1 (78.1%). On the other hand, tumors were as a rule negative to CD117 (95.0%), CK5 (90.0%), p53 (83.3%) and p63 (95.4%). Cyclin E1 negativity was noted in nearly half of cases. p21 low expression (≤10%) was noted in 76.1% of cases, whereas p27 high expression (>50%) was observed in 62.2%.

S2 Table presents the associations between expression of markers and patient/disease characteristics. Cyclin D1 positive cases were predominantly ER positive and PgR positive. Luminal B cases presented with the higher levels of cyclin D1 expression (87.3%), whereas the lowest levels of cyclin D1 expression were noted among TNBC patients (31.9%). The highest level of CD117 positivity was noted among TNBC cases (13.2%). CK5 positive expression correlated with higher histological grade and TNBC phenotype. Cyclin E1 negative expression correlated with ER positive status, whereas TNBCs were more frequently positive to cyclin E1. p21 high expression correlated with HER2-enriched subtype (10.2%). p27 low and moderate expression was associated with higher histological grade, while high p27 expression correlated with the Luminal B subtype. p53 immunopositivity was associated with high histological grade, while absence of p53 expression with the Luminal A subtype (96.6%). p63 expression was associated with absence of lymphatic vessel invasion. Of note, there was no correlation between different treatment groups and protein expression.

The inter-correlations between the examined markers are depicted in S3 Table. Patients positive to cyclin D1 were more likely to be negative to CK5, express a higher amount of p21, express a higher amount of p27 and to be p53-negative. CD117 negative cases were also negative to CK5, while CK5 positive cases were significantly more frequently cyclin E1-positive. CK5 negative patients were more frequently p53- and p63-negative and expressed higher levels of p27. Cyclin E1-positive cases expressed lower levels of p21. p63-negative cases expressed low level p21, whereas p53-negative cases expressed high p27.

### Survival

Median follow-up was 121.1 months (range 0.5–191.9 months. Until the last date of follow-up, 388 patients (36.0%) experienced disease relapse with 5-year DFS rate of 74.1%, while 308 patients (28.6%) died with 5-year OS rate of 86.1%. Median DFS was 184.7 months while median OS was not reached yet.

#### Hierarchical clustering analysis

**Fig 3:**
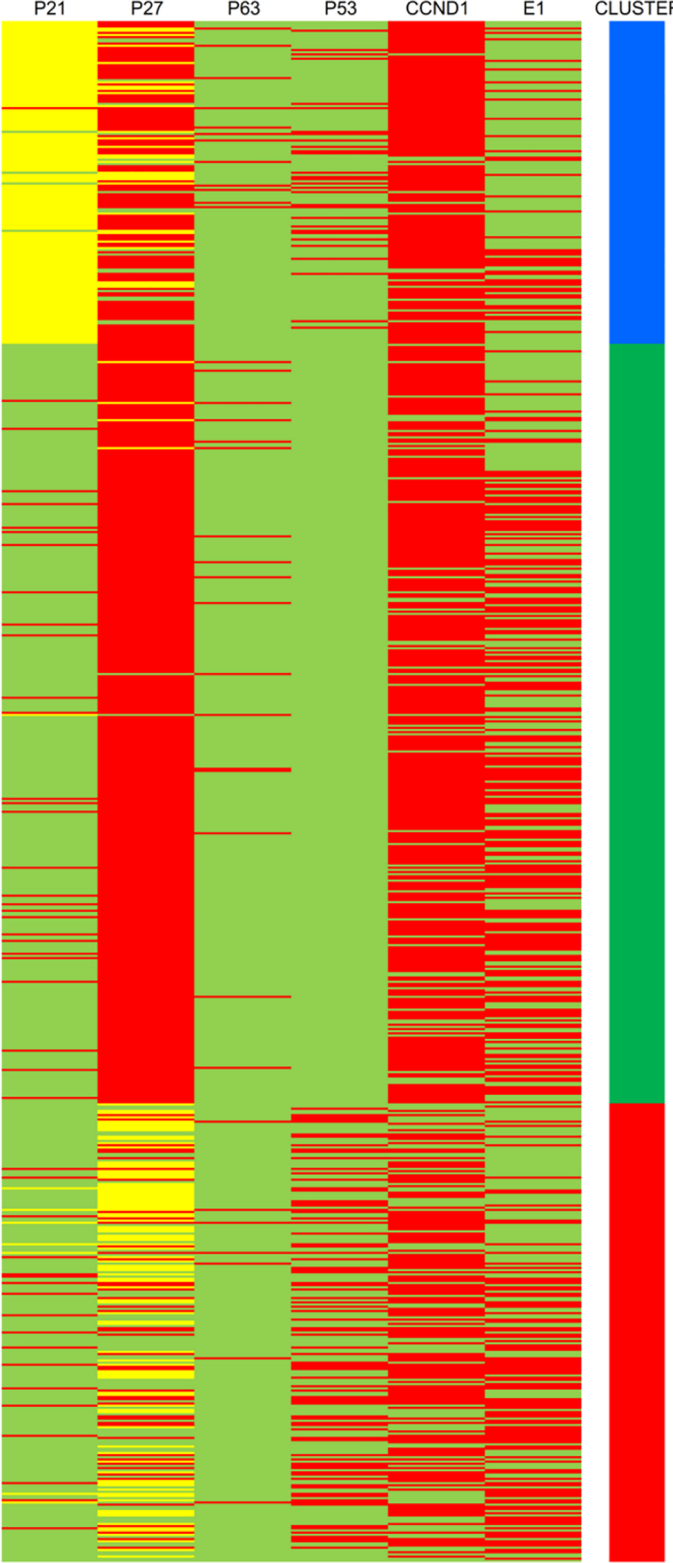
Heatmap of the resulted clusters (green: low or negative, yellow: moderate, red: positive expression; blue: cluster 1, green: cluster 2, red: cluster 3).

**Fig 4:**
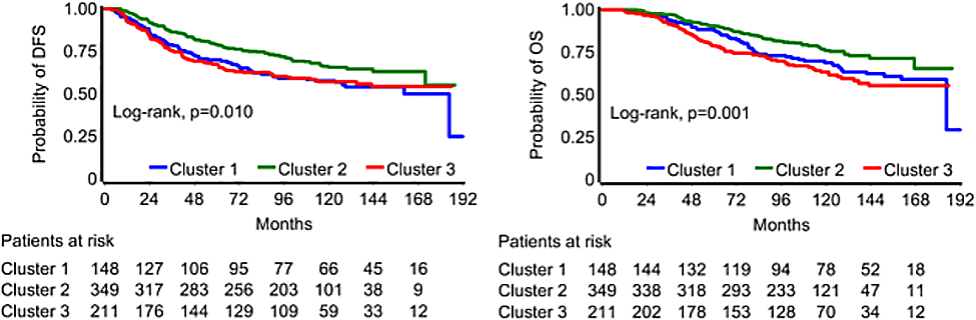
Kaplan-Meier curves for DFS and OS for clusters.

Tumors in cluster 1 (N = 150) were positive for cyclin D1 and/or E1 but moderately expressed p21, p27 and p53. Tumors in cluster 2 (N = 353) did not express p53 protein but were all positive for cyclin D1 and/or cyclin E1, and for p27. Lastly, tumors in cluster 3 (N = 213) were negative or showed moderate expression of p21 and p27, but were positive for cyclin D1 or E1 and p53 expression (Fig 3). Tumors in cluster 2 had the lowest proliferation rate (Ki67 median-value 20, range 0–95), as compared to tumors in clusters 1 and 3 (Ki67 median-value 30, range 1–95; Kruskal- Wallis p<0.001). Additionally, a significant association between cluster membership and ER/PgR status, HER2 status, basal phenotype and breast cancer subtypes was observed (Chi-square, all p-values <0.001). More specifically, ER/PgR positive tumors (291 out of 523, 55.6%) and HER2 negative tumors (264 out of 480, 55%) were more frequent in cluster 2. Finally, the majority of basal-like tumors (49 out of 96, 51%) and TNBC (34 out of 55, 61.8%) were in cluster 3. No other meaningful association was observed between cluster membership and clinicopathological parameters. The 5-year DFS and OS rates for clusters 1, 2 and 3 were 70.0%, 79.1%, 67.4% and 88.4%, 90.4%, 78.9%, respectively. Cluster 2 appeared to have better prognosis compared to clusters 1 and 3 (OS log-rank p-value = 0.001; DFS p-value = 0.010, Fig 4).

#### Multivariate analysis

Table 2 presents the results of the multivariate Cox regression analysis. After adjustment for clinical factors, the clustering scheme remained significant regarding OS (Wald’s p = 0.006) but not DFS (p = 0.145). Specifically, cluster 2 showed better OS compared to cluster 1 (adjusted HR = 0.60, 95% CI 0.43–0.82, p = 0.002). Moreover, Ki67 expression was associated with a decrease in OS (p = 0.005). Lower number of positive lymph nodes was associated with better OS (adjusted HR = 0.40, 95% CI 0.29–0.57, p<0.001) and better DFS (adjusted HR = 0.50, 95% CI 0.38–0.67, p<0.001).

**Table 2:** Results of the multivariate Cox regression analyses.

**Disease-free survival** **(N = 695)**	**Number of patients**	**Number of events**	**Hazard ratio**	**95% CIs**	**Wald's p**
Surgery					
Partial/Simple mastectomy vs. Modified radical	209 vs. 486	62 vs. 202	0.72	0.54–0.97	0.030
Tumor size					
>2 vs. ≤2	469 vs. 226	192 vs. 72	1.25	0.95–1.64	0.12
Number of positive nodes					
1–3 vs. ≥4	428 vs. 267	199 vs. 65	0.5	0.38–0.67	<0.001
Subtypes					*0*.*035*
Luminal A vs. TNBC	296 vs. 164	129 vs. 40	0.51	0.31–0.84	0.008
Luminal B vs. TNBC	109 vs. 164	43 vs. 40	0.86	0.57–1.30	0.48
Luminal-HER2 vs. TNBC	62 vs. 164	21 vs. 40	0.81	0.50–1.30	0.37
HER2-Enriched vs. TNBC	64 vs. 164	31 vs. 40	0.65	0.37–1.14	0.13
Cluster					*0*.*145*
1 vs. 3	344 vs. 141	110 vs. 65	0.90	0.65–1.26	0.56
2 vs. 3	210 vs. 141	89 vs. 65	0.75	0.56–1.01	0.055
**Overall survival** **(N = 701)**	**Number of patients**	**Number of events**	**Hazard ratio**	**95% CIs**	**Wald's p**
Surgery					
Partial/Simple mastectomy vs. Modified radical	209 vs. 492	48 vs. 161	0.76	0.55–1.06	0.11
Tumor size					
>2 vs. ≤2	473 vs. 228	157 vs. 52	1.39	1.01–1.91	0.041
Number of positive nodes					
1–3 vs. ≥4	431 vs. 270	167 vs. 42	0.4	0.29–0.57	<0.001
Cluster					*0*.*006*
1 vs. 3	345 vs. 147	77 vs. 53	0.73	0.51–1.04	0.077
2 vs. 3	209 vs. 147	79 vs. 53	0.60	0.43–0.82	0.002
Ki67 (increase by 5%)			59.27	3.48–1009	0.005

## Discussion

This study highlights the original concept of protein expression patterns with special prognostic relevance in the context of early breast cancer. Three clusters emerged from our elaborate analysis; the most favorable of them (cluster 2) was the one characterized by lack of p53 protein expression in parallel with the expression of the tumor suppressor protein p27. On the other hand, cluster 1, whose distinct feature pertained to the moderate expression of p21, did not seem to differ from the burdened cluster 3. The latter was characterized by the poor prognostic aspect of positive p53 status [26], often accompanied by negativity of the tumor suppressor proteins p21 and p27 [27]. Of note, cyclin D1 and E1 both correlated with better outcome, either singly or clustered; this finding may seem counterintuitive as CCND1 is a weak oncogene, but is in line with earlier studies showing that over-expression of cyclin D1 is in fact associated with favorable outcomes in breast cancer, both in terms of prognosis and response to endocrine treatment [28, 29]. Interestingly enough, in a recently published meta-analysis, the impact of CCND1 expression on OS was a 1.67-fold increased risk for patients with ER-positive breast cancer; however, according to this meta-analysis, CCND1 overexpression does not impact the prognosis of patients with unselected primary breast cancer [30].

Apart from the prognostic relevance of the aforementioned clusters, this study provides interesting insight into aspects of the molecular network, in relation to the clinicopathological features of patients. Luminal B cases presented with the highest levels of cyclin D1 expression, a fact that has proven useful in the advent of CDK4/6 inhibitors [9]; these agents are FDA approved and are currently evaluated in the context of clinical trials on Luminal B and HER2-negative subtypes [9, 31]. Interestingly enough, Li et al [32], have reported an optimal cut-off point of immunostaining scores of cyclin D1 protein, which could be used to predict the status of CCND1 gene and identify a subgroup of ER positive breast cancers with poor response to endocrine agents.

The intense expression of cyclin E1 in TNBC seems rather rational, considering it as part of the more aggressive phenotype of the latter subtype; this finding seems in line with the study by Agarwal et al [33], which indicated that cyclin E1 gene was present in significantly higher copy numbers in basal-like versus other breast cancer subtypes. Accordingly, Luhtala et al [34], have been reported that cyclin E is frequently over-expressed but with limited prognostic or predictive value in HER-2-positive breast cancer irrespectively of trastuzumab therapy.

In our study, p27 low expression was associated with higher histological grade, a finding that is in accordance with the existing literature [35]. Of note, several studies have shown that in breast cancer cells, the p27 expression level usually decreases during progression of the disease [36]. Moreover, it has been found that its phosphorylation status is the key regulator and that several signal transduction pathways are involved in the regulation of both the expression and distribution of p27 [36]. On the other hand, in our series, high p21 expression correlated with HER2-enriched subtype; this observation does not seem in agreement with other studies, which supported that the expression of p21 protein was higher in HER2-negative as compared to positive tumors [37, 38]. Furthermore, regarding the validity of our approach, anticipated associations, such as the one between p53 immunopositivity and high histological grade, were identified in our sample. Accordingly, Luminal A cases, which are characterized by best prognosis, were negative for p53. Of note, Watanabe et al [39], have found that p53 mutant-like is an independent prognostic factor in the multivariate analysis in breast cancer patients and seems to be a strong prognostic factor that could identify patients with a poorer prognosis.

Regarding the inter-correlations between the examined molecules-markers, a few associations seem worth commenting. For instance, the association between cyclin D1 and higher expression of p27 does not seem paradoxical, as it has been previously described in cell lines [40]. Moreover, it has been reported that breast as well as ovarian cancers often had cells that co-expressed the p21 and cyclin D1 genes, often leading to growth arrest [41]. Moreover, it has been shown that cyclin D1 cooperates with p21 to regulate TGFβ-mediated breast cancer cell migration and tumor local invasion [42]. Hence, co-expression of cell cycle inducers and inhibitors may indicate that key aspects of canonical cell cycle regulation are retained in these tumors. In fact, the favorable prognostic effects of cyclin D1 and E1 expression can be explained under this prism [23, 24]. Of note, other researchers have found that concomitant inactivation of the p53 and pRB functional pathways predict resistance towards DNA damaging agents (anthracyclines and mitomycin) in breast cancer in vivo [43].

Despite the originality of the present protein expression patterns, this study bears certain limitations that should be addressed and discussed. First, analytical limitations of immunohistochemistry as a method may have not allowed for a more precise distinction of protein expression levels. In addition, our findings are not backed by mRNA expression or genomic data, which would provide a more complete picture of the altered molecular status in tumors with respect to cell cycle checkpoint defects. Moreover, the treatment protocols administered to patients did not include trastuzumab given the study period prior to the introduction of trastuzumab in the adjuvant setting. Missing values were sometimes present in the analyses due to occasional lack of tissue for the relevant analyses; nevertheless, this non-availability was non-systematic, spanning the whole database of included clinical trials. On the other hand, among the strengths of this study, patients come from the well-defined set of HeCOG breast cancer registered clinical trials, whereas the considerable sample size should also be underlined. Furthermore, the network of examined, numerous molecules yielded a rather rich and clear picture of the possible underlying associations.

In conclusion, this sizable study presents a novel set of three molecular clusters, which seem to entail a significant prognostic value. The protein expression patterns of the examined cell cycle regulators seem to critically cooperate with p53 status, concerting the modification of prognosis in operable breast cancer. The present study findings may prove useful in the clinical applicability of CDK4/6 inhibitors on breast cancer patients, according to molecular profile of the tumors.

## Supporting information

S1 Table Clinical trial characteristics. (DOC)

S2 Table Associations of the examined markers with patient and tumor characteristics—N (%). Bold cells indicate p-values lower than 0.05.
(DOC)

S3 Table Associations among the examined markers—N (%). (DOC)
