# Supplementary material for: Protein expression patterns of cell cycle regulators in operable breast cancer
Source: PLoS One. 2017 Aug 10;12(8):e0180489. doi: 10.1371/journal.pone.0180489 (PMC5552326; doi:10.1371/journal.pone.0180489)
Supplement: S2 Table — Bold cells indicate p-values lower than 0.05. (DOC) [file pone.0180489.s002.doc]

**S2 Table.** Associations of the examined markers with patient and tumor characteristics - N (%). Bold cells indicate p-values lower than 0.05.

|  |  | **CCND1** | | | **CD117** | | | **CK5** | | | **Cyclin E1** | | | **P21** | | | | **P27** | | | | **P53** | | | **P63** | | |
| --- | --- | --- | --- | --- | --- | --- | --- | --- | --- | --- | --- | --- | --- | --- | --- | --- | --- | --- | --- | --- | --- | --- | --- | --- | --- | --- | --- |
|  |  | **Positive** | **Negative** | **P-value** | **Positive** | **Negative** | **P-value** | **Positive** | **Negative** | **P-value** | **Positive** | **Negative** | **P-value** | **≤10** | **11-50** | **>50** | **P-value** | **≤10** | **11-50** | **>50** | **P-value** | **Positive** | **Negative** | **P-value** | **Positive** | **Negative** | **P-value** |
|  |  |  |  |  |  |  |  |  |  |  |  |  |  |  |  |  |  |  |  |  |  |  |  |  |  |  |  |
| Menopausal status | Peri | 10 (76.9) | 3 (23.1) | 0.97 | 2 (9.1) | 20 (90.9) | 0.658 | 3 (13.6) | 19 (86.4) | 0.805 | 12 (60.0) | 8 (40.0) | 0.399 | 18 (90.0) | 2 (10.0) | 0 (0.0) | 0.124 | 5 (26.3) | 4 (21.1) | 10 (52.6) | 0.467 | 6 (27.3) | 16 (72.7) | 0.321 | 0 (0.0) | 20 (100.0) | 0.493 |
|  | Post | 343 (77.8) | 98 (22.2) |  | 28 (5.1) | 524 (94.9) |  | 53 (9.6) | 497 (90.4) |  | 227 (45.3) | 274 (54.7) |  | 408 (77.4) | 88 (16.7) | 31 (5.9) |  | 108 (21.3) | 95 (18.7) | 305 (60.0) |  | 94 (17.1) | 455 (82.9) |  | 27 (5.1) | 504 (94.9) |  |
|  | Pre | 309 (78.4) | 85 (21.6) |  | 22 (4.8) | 441 (95.2) |  | 46 (10.2) | 403 (89.8) |  | 196 (47.2) | 219 (52.8) |  | 326 (73.9) | 96 (21.8) | 19 (4.3) |  | 74 (18.0) | 68 (16.6) | 268 (65.4) |  | 69 (15.5) | 375 (84.5) |  | 19 (4.2) | 433 (95.8) |  |
|  |  |  |  |  |  |  |  |  |  |  |  |  |  |  |  |  |  |  |  |  |  |  |  |  |  |  |  |
| Histological grade | 1-2 | 350 (80.5) | 85 (19.5) | 0.071 | 25 (4.8) | 498 (95.2) | 0.70 | 29 (5.7) | 478 (94.3) | **<0.001** | 202 (44.2) | 255 (55.8) | 0.197 | 381 (77.1) | 92 (18.6) | 21 (4.3) | 0.454 | 77 (16.7) | 67 (14.5) | 317 (68.8) | **<0.001** | 54 (10.8) | 445 (89.2) | **<0.001** | 26 (5.3) | 468 (94.7) | 0.326 |
|  | 3-4 | 308 (75.3) | 101 (24.7) |  | 27 (5.3) | 482 (94.7) |  | 73 (14.3) | 437 (85.7) |  | 230 (48.4) | 245 (51.6) |  | 366 (74.8) | 94 (19.2) | 29 (5.9) |  | 109 (23.1) | 99 (21.0) | 263 (55.8) |  | 115 (22.5) | 397 (77.5) |  | 20 (4.0) | 485 (96.0) |  |
|  |  |  |  |  |  |  |  |  |  |  |  |  |  |  |  |  |  |  |  |  |  |  |  |  |  |  |  |
| Histological type | Ductal | 531 (78.3) | 147 (21.7) | **0.002** | 40 (4.8) | 794 (95.2) | **0.016** | 85 (10.4) | 736 (89.6) | **<0.001** | 356 (46.8) | 404 (53.2) | 0.969 | 599 (75.7) | 146 (18.5) | 46 (5.8) | 0.425 | 156 (20.6) | 134 (17.7) | 466 (61.6) | 0.063 | 142 (17.4) | 676 (82.6) | **0.001** | 31 (3.8) | 776 (96.2) | 0.077 |
|  | Inflammatory | 2 (100.0) |  |  | 1 (16.7) | 5 (83.3) |  | 1 (16.7) | 5 (83.3) |  | 2 (50.0) | 2 (50.0) |  | 3 (75.0) | 1 (25.0) | 0 (0.0) |  | 1 (20.0) | 2 (40.0) | 2 (40.0) |  | 3 (50.0) | 3 (50.0) |  | 0 (0.0) | 5 (100.0) |  |
|  | Lobular | 69 (80.2) | 17 (19.8) |  | 5 (4.7) | 101 (95.3) |  | 5 (4.7) | 102 (95.3) |  | 42 (46.7) | 48 (53.3) |  | 85 (81.7) | 18 (17.3) | 1 (1.0) |  | 14 (14.9) | 14 (14.9) | 66 (70.2) |  | 6 (5.8) | 98 (94.2) |  | 8 (7.9) | 93 (92.1) |  |
|  | Mixed | 58 (79.5) | 15 (20.5) |  | 3 (3.8) | 76 (96.2) |  | 5 (6.7) | 70 (93.3) |  | 30 (42.9) | 40 (57.1) |  | 55 (72.4) | 19 (25.0) | 2 (2.6) |  | 12 (16.9) | 12 (16.9) | 47 (66.2) |  | 13 (17.3) | 62 (82.7) |  | 5 (6.4) | 73 (93.6) |  |
|  | Other | 2 (22.2) | 7 (77.8) |  | 3 (25.0) | 9 (75.0) |  | 6 (50.0) | 6 (50.0) |  | 5 (41.7) | 7 (58.3) |  | 10 (76.9) | 2 (15.4) | 1 (7.7) |  | 4 (36.4) | 5 (45.5) | 2 (18.2) |  | 5 (41.7) | 7 (58.3) |  | 2 (16.7) | 10 (83.3) |  |
|  |  |  |  |  |  |  |  |  |  |  |  |  |  |  |  |  |  |  |  |  |  |  |  |  |  |  |  |
| Tumor size | ≤2 | 223 (81.4) | 51 (18.6) | 0.106 | 20 (6.0) | 311 (94.0) | 0.299 | 29 (9.0) | 295 (91.0) | 0.45 | 143 (49.0) | 149 (51.0) | 0.302 | 231 (75.2) | 61 (19.9) | 15 (4.9) | 0.848 | 51 (17.7) | 54 (18.8) | 183 (63.5) | 0.504 | 52 (16.4) | 266 (83.6) | 0.863 | 15 (4.8) | 295 (95.2) | 0.798 |
|  | >2 | 439 (76.5) | 135 (23.5) |  | 32 (4.5) | 674 (95.5) |  | 73 (10.5) | 624 (89.5) |  | 292 (45.3) | 352 (54.7) |  | 521 (76.5) | 125 (18.4) | 35 (5.1) |  | 136 (21.0) | 113 (17.4) | 400 (61.6) |  | 117 (16.8) | 580 (83.2) |  | 31 (4.5) | 662 (95.5) |  |
|  |  |  |  |  |  |  |  |  |  |  |  |  |  |  |  |  |  |  |  |  |  |  |  |  |  |  |  |
| Number of positive nodes | 1-3 | 266 (79.6) | 68 (20.4) | 0.37 | 20 (4.8) | 401 (95.2) | 0.75 | 40 (9.7) | 372 (90.3) | 0.805 | 184 (49.6) | 187 (50.4) | 0.121 | 320 (81.4) | 51 (13.0) | 22 (5.6) | **0.001** | 91 (24.1) | 59 (15.6) | 227 (60.2) | **0.022** | 57 (14.0) | 350 (86.0) | 0.064 | 14 (3.5) | 386 (96.5) | 0.18 |
|  | ≥4 | 396 (77.0) | 118 (23.0) |  | 32 (5.2) | 584 (94.8) |  | 62 (10.2) | 547 (89.8) |  | 251 (44.4) | 314 (55.6) |  | 432 (72.6) | 135 (22.7) | 28 (4.7) |  | 96 (17.1) | 108 (19.3) | 356 (63.6) |  | 112 (18.4) | 496 (81.6) |  | 32 (5.3) | 571 (94.7) |  |
|  |  |  |  |  |  |  |  |  |  |  |  |  |  |  |  |  |  |  |  |  |  |  |  |  |  |  |  |
| Surgery (grouped) | Modified radical | 468 (78.8) | 126 (21.2) | 0.437 | 26 (3.7) | 677 (96.3) | **0.004** | 57 (8.2) | 639 (91.8) | **0.004** | 281 (43.8) | 360 (56.2) | **0.013** | 513 (75.3) | 135 (19.8) | 33 (4.8) | 0.484 | 115 (17.8) | 124 (19.2) | 406 (62.9) | **0.034** | 110 (16.0) | 579 (84.0) | 0.371 | 31 (4.5) | 654 (95.5) | 0.876 |
|  | Partial/Simple mastectomy | 194 (76.4) | 60 (23.6) |  | 26 (7.8) | 306 (92.2) |  | 45 (13.9) | 278 (86.1) |  | 154 (52.6) | 139 (47.4) |  | 237 (77.7) | 51 (16.7) | 17 (5.6) |  | 71 (24.5) | 43 (14.8) | 176 (60.7) |  | 59 (18.2) | 265 (81.8) |  | 15 (4.7) | 301 (95.3) |  |
|  |  |  |  |  |  |  |  |  |  |  |  |  |  |  |  |  |  |  |  |  |  |  |  |  |  |  |  |
| Lymph invasion | Yes | 275 (79.7) | 70 (20.3) | 0.48 | 18 (4.0) | 434 (96.0) | **0.034** | 35 (7.8) | 414 (92.2) | 0.096 | 223 (55.1) | 182 (44.9) | 0.727 | 343 (82.3) | 46 (11.0) | 28 (6.7) | 0.464 | 76 (18.9) | 51 (12.7) | 275 (68.4) | **0.008** | 75 (16.9) | 370 (83.1) | 0.098 | 6 (1.4) | 420 (98.6) | **0.018** |
|  | No | 205 (77.4) | 60 (22.6) |  | 26 (7.4) | 325 (92.6) |  | 38 (11.3) | 299 (88.7) |  | 165 (53.7) | 142 (46.3) |  | 270 (82.3) | 42 (12.8) | 16 (4.9) |  | 88 (28.6) | 40 (13.0) | 180 (58.4) |  | 42 (12.6) | 292 (87.4) |  | 14 (4.2) | 321 (95.8) |  |
|  |  |  |  |  |  |  |  |  |  |  |  |  |  |  |  |  |  |  |  |  |  |  |  |  |  |  |  |
| ER status | Positive | 569 (85.2) | 99 (14.8) | **<0.001** | 25 (3.3) | 740 (96.7) | **<0.001** | 21 (2.8) | 727 (97.2) | **<0.001** | 298 (43.8) | 383 (56.2) | **0.006** | 548 (76.2) | 136 (18.9) | 35 (4.9) | 0.901 | 91 (13.5) | 94 (13.9) | 490 (72.6) | **<0.001** | 81 (10.9) | 659 (89.1) | **<0.001** | 30 (4.1) | 706 (95.9) | 0.20 |
|  | Negative | 93 (51.7) | 87 (48.3) |  | 27 (9.9) | 245 (90.1) |  | 81 (29.7) | 192 (70.3) |  | 137 (53.7) | 118 (46.3) |  | 204 (75.8) | 50 (18.6) | 15 (5.6) |  | 96 (36.6) | 73 (27.9) | 93 (35.5) |  | 88 (32.0) | 187 (68.0) |  | 16 (6.0) | 251 (94.0) |  |
|  |  |  |  |  |  |  |  |  |  |  |  |  |  |  |  |  |  |  |  |  |  |  |  |  |  |  |  |
| PgR status | Positive | 518 (84.4) | 96 (15.6) | **<0.001** | 25 (3.6) | 675 (96.4) | **0.002** | 26 (3.8) | 662 (96.2) | **<0.001** | 279 (44.6) | 347 (55.4) | 0.097 | 508 (77.2) | 122 (18.5) | 28 (4.3) | 0.232 | 85 (13.7) | 83 (13.4) | 453 (72.9) | **<0.001** | 66 (9.7) | 616 (90.3) | **<0.001** | 25 (3.7) | 652 (96.3) | 0.051 |
|  | Negative | 144 (61.5) | 90 (38.5) |  | 27 (8.0) | 310 (92.0) |  | 76 (22.8) | 257 (77.2) |  | 156 (50.3) | 154 (49.7) |  | 244 (73.9) | 64 (19.4) | 22 (6.7) |  | 102 (32.3) | 84 (26.6) | 130 (41.1) |  | 103 (30.9) | 230 (69.1) |  | 21 (6.4) | 305 (93.6) |  |
|  |  |  |  |  |  |  |  |  |  |  |  |  |  |  |  |  |  |  |  |  |  |  |  |  |  |  |  |
| ERPgR status | Positive | 590 (84.5) | 108 (15.5) | **<0.001** | 28 (3.5) | 778 (96.5) | **<0.001** | 32 (4.1) | 758 (95.9) | **<0.001** | 313 (43.4) | 409 (56.6) | **<0.001** | 583 (76.7) | 142 (18.7) | 35 (4.6) | 0.463 | 99 (13.8) | 107 (14.9) | 510 (71.2) | **<0.001** | 85 (10.9) | 697 (89.1) | **<0.001** | 35 (4.5) | 743 (95.5) | 0.805 |
|  | Negative | 72 (48.0) | 78 (52.0) |  | 24 (10.4) | 207 (89.6) |  | 70 (30.3) | 161 (69.7) |  | 122 (57.0) | 92 (43.0) |  | 169 (74.1) | 44 (19.3) | 15 (6.6) |  | 88 (39.8) | 60 (27.1) | 73 (33.0) |  | 84 (36.1) | 149 (63.9) |  | 11 (4.9) | 214 (95.1) |  |
|  |  |  |  |  |  |  |  |  |  |  |  |  |  |  |  |  |  |  |  |  |  |  |  |  |  |  |  |
| Subtypes | LuminalA | 173 (84.0) | 33 (16.0) | **<0.001** | 10 (4.0) | 239 (96.0) | **<0.001** | 5 (2.0) | 240 (98.0) | **<0.001** | 98 (45.4) | 118 (54.6) | **0.027** | 213 (93.4) | 11 (4.8) | 4 (1.8) | **<0.001** | 38 (17.4) | 26 (11.9) | 154 (70.6) | **<0.001** | 8 (3.4) | 229 (96.6) | **<0.001** | 10 (4.3) | 224 (95.7) | 0.768 |
|  | Luminal B | 295 (87.3) | 43 (12.7) |  | 14 (3.6) | 370 (96.4) |  | 21 (5.5) | 364 (94.5) |  | 153 (42.6) | 206 (57.4) |  | 254 (68.1) | 96 (25.7) | 23 (6.2) |  | 43 (12.2) | 51 (14.5) | 258 (73.3) |  | 42 (10.9) | 342 (89.1) |  | 18 (4.7) | 363 (95.3) |  |
|  | Luminal-HER2 | 97 (79.5) | 25 (20.5) |  | 3 (2.2) | 134 (97.8) |  | 5 (3.7) | 131 (96.3) |  | 58 (46.4) | 67 (53.6) |  | 96 (75.0) | 24 (18.8) | 8 (6.3) |  | 16 (13.2) | 23 (19.0) | 82 (67.8) |  | 33 (24.4) | 102 (75.6) |  | 3 (2.2) | 131 (97.8) |  |
|  | HER2 enriched | 46 (61.3) | 29 (38.7) |  | 8 (7.6) | 97 (92.4) |  | 13 (12.5) | 91 (87.5) |  | 53 (55.8) | 42 (44.2) |  | 65 (66.3) | 23 (23.5) | 10 (10.2) |  | 37 (37.4) | 25 (25.3) | 37 (37.4) |  | 36 (35.3) | 66 (64.7) |  | 5 (4.9) | 98 (95.1) |  |
|  | TNBC | 23 (31.9) | 49 (68.1) |  | 16 (13.2) | 105 (86.8) |  | 57 (46.7) | 65 (53.3) |  | 66 (57.4) | 49 (42.6) |  | 101 (81.5) | 19 (15.3) | 4 (3.2) |  | 49 (41.9) | 34 (29.1) | 34 (29.1) |  | 47 (37.3) | 79 (62.7) |  | 6 (5.2) | 110 (94.8) |  |
|  |  |  |  |  |  |  |  |  |  |  |  |  |  |  |  |  |  |  |  |  |  |  |  |  |  |  |  |
| Treatment group | HE1000-A: E-T-CMF | 210 (72.9) | 78 (27.1) | 0.055 | 24 (6.3) | 355 (93.7) | 0.072 | 37 (10.1) | 331 (89.9) | 0.66 | 196 (59.0) | 136 (41.0) | **<0.001** | 294 (83.5) | 31 (8.8) | 27 (7.7) | **<0.001** | 75 (22.6) | 39 (11.7) | 218 (65.7) | **<0.001** | 45 (12.3) | 321 (87.7) | **0.016** | 9 (2.5) | 349 (97.5) | **<0.001** |
|  | HE1000-B: ET-CMF | 224 (82.4) | 48 (17.6) |  | 22 (5.8) | 355 (94.2) |  | 34 (9.1) | 339 (90.9) |  | 189 (55.8) | 150 (44.2) |  | 303 (87.8) | 27 (7.8) | 15 (4.3) |  | 83 (24.8) | 40 (11.9) | 212 (63.3) |  | 63 (17.2) | 304 (82.8) |  | 8 (2.3) | 347 (97.7) |  |
|  | HE1097-A: E-T-CMF | 108 (79.4) | 28 (20.6) |  | 2 (1.5) | 129 (98.5) |  | 12 (9.2) | 118 (90.8) |  | 24 (20.3) | 94 (79.7) |  | 73 (55.3) | 56 (42.4) | 3 (2.3) |  | 15 (12.5) | 42 (35.0) | 63 (52.5) |  | 27 (20.6) | 104 (79.4) |  | 12 (9.0) | 122 (91.0) |  |
|  | HE1097-B: E-CMF | 120 (78.9) | 32 (21.1) |  | 4 (2.7) | 146 (97.3) |  | 19 (12.7) | 131 (87.3) |  | 26 (17.7) | 121 (82.3) |  | 82 (51.6) | 72 (45.3) | 5 (3.1) |  | 14 (9.3) | 46 (30.7) | 90 (60.0) |  | 34 (22.5) | 117 (77.5) |  | 17 (10.9) | 139 (89.1) |  |
