# Supplementary material for: Protein expression patterns of cell cycle regulators in operable breast cancer
Source: PLoS One. 2017 Aug 10;12(8):e0180489. doi: 10.1371/journal.pone.0180489 (PMC5552326; doi:10.1371/journal.pone.0180489)
Supplement: S3 Table — (DOC) [file pone.0180489.s003.doc]

**S3 Table.** Associations among the examined markers - N (%)

|  |  | **CCND1** | | | **CD117** | | | **CK5** | | | **Cyclin E1** | | | **P21** | | | | **P27** | | | | **P53** | | |
| --- | --- | --- | --- | --- | --- | --- | --- | --- | --- | --- | --- | --- | --- | --- | --- | --- | --- | --- | --- | --- | --- | --- | --- | --- |
|  |  | **Positive** | **Negative** | **P-value** | **Positive** | **Negative** | **P-value** | **Positive** | **Negative** | **P-value** | **Positive** | **Negative** | **P-value** | **≤10** | **11-50** | **>50** | **P-value** | **≤10** | **11-50** | **>50** | **P-value** | **Positive** | **Negative** | **P-value** |
|  |  |  |  |  |  |  |  |  |  |  |  |  |  |  |  |  |  |  |  |  |  |  |  |  |
| CD117 | Negative | 631 (96.6) | 167 (94.9) | 0.28 |  |  |  |  |  |  |  |  |  |  |  |  |  |  |  |  |  |  |  |  |
|  | Positive | 22 (3.4) | 9 (5.1) |  |  |  |  |  |  |  |  |  |  |  |  |  |  |  |  |  |  |  |  |  |
|  |  |  |  |  |  |  |  |  |  |  |  |  |  |  |  |  |  |  |  |  |  |  |  |  |
| CK5 | Negative | 615 (97.0) | 135 (76.7) | **<0.001** | 36 (72.0) | 864 (91.5) | **<0.001** |  |  |  |  |  |  |  |  |  |  |  |  |  |  |  |  |  |
|  | Positive | 19 (3.0) | 41 (23.3) |  | 14 (28.0) | 80 (8.5) |  |  |  |  |  |  |  |  |  |  |  |  |  |  |  |  |  |  |
|  |  |  |  |  |  |  |  |  |  |  |  |  |  |  |  |  |  |  |  |  |  |  |  |  |
| Cyclin E1 | Negative | 340 (56.0) | 83 (49.1) | 0.11 | 21 (45.7) | 469 (53.7) | 0.29 | 36 (39.1) | 444 (54.9) | **0.004** |  |  |  |  |  |  |  |  |  |  |  |  |  |  |
|  | Positive | 267 (44.0) | 86 (50.9) |  | 25 (54.3) | 404 (46.3) |  | 56 (60.9) | 365 (45.1) |  |  |  |  |  |  |  |  |  |  |  |  |  |  |  |
|  |  |  |  |  |  |  |  |  |  |  |  |  |  |  |  |  |  |  |  |  |  |  |  |  |
| P21 | ≤10 | 445 (70.4) | 148 (82.2) | **0.003** | 45 (90.0) | 693 (75.7) | 0.056 | 69 (74.2) | 652 (76.5) | 0.87 | 338 (80.3) | 351 (72.1) | **0.001** |  |  |  |  |  |  |  |  |  |  |  |
|  | 11-50 | 150 (23.7) | 22 (12.2) |  | 3 (6.0) | 174 (19.0) |  | 19 (20.4) | 156 (18.3) |  | 57 (13.5) | 114 (23.4) |  |  |  |  |  |  |  |  |  |  |  |  |
|  | >50 | 37 (5.9) | 10 (5.6) |  | 2 (4.0) | 48 (5.2) |  | 5 (5.4) | 44 (5.2) |  | 26 (6.2) | 22 (4.5) |  |  |  |  |  |  |  |  |  |  |  |  |
|  |  |  |  |  |  |  |  |  |  |  |  |  |  |  |  |  |  |  |  |  |  |  |  |  |
| P27 | ≤10 | 50 (8.3) | 53 (30.6) | **<0.001** | 14 (28.6) | 168 (19.4) | 0.252 | 34 (36.6) | 149 (18.5) | **<0.001** | 85 (20.5) | 89 (18.7) | 0.36 | 152 (22.0) | 24 (13.6) | 7 (14.0) | 0.11 |  |  |  |  |  |  |  |
|  | 11-50 | 90 (14.9) | 45 (26.0) |  | 9 (18.4) | 151 (17.4) |  | 35 (37.6) | 119 (14.7) |  | 67 (16.1) | 94 (19.7) |  | 119 (17.2) | 34 (19.2) | 10 (20.0) |  |  |  |  |  |  |  |  |
|  | >50 | 464 (76.8) | 75 (43.4) |  | 26 (53.1) | 548 (63.2) |  | 24 (25.8) | 539 (66.8) |  | 263 (63.4) | 293 (61.6) |  | 420 (60.8) | 119 (67.2) | 33 (66.0) |  |  |  |  |  |  |  |  |
|  |  |  |  |  |  |  |  |  |  |  |  |  |  |  |  |  |  |  |  |  |  |  |  |  |
| P53 | Negative | 551 (86.9) | 134 (75.7) | **<0.001** | 39 (75.0) | 783 (83.7) | 0.10 | 60 (58.8) | 770 (86.2) | **<0.001** | 344 (81.1) | 405 (83.9) | 0.28 | 605 (83.8) | 144 (81.8) | 40 (81.6) | 0.78 | 147 (80.8) | 113 (72.4) | 491 (86.6) | **<0.001** |  |  |  |
|  | Positive | 83 (13.1) | 43 (24.3) |  | 13 (25.0) | 153 (16.3) |  | 42 (41.2) | 123 (13.8) |  | 80 (18.9) | 78 (16.1) |  | 117 (16.2) | 32 (18.2) | 9 (18.4) |  | 35 (19.2) | 43 (27.6) | 76 (13.4) |  |  |  |  |
|  |  |  |  |  |  |  |  |  |  |  |  |  |  |  |  |  |  |  |  |  |  |  |  |  |
| P63 | Negative | 618 (95.4) | 164 (93.2) | 0.24 | 48 (96.0) | 895 (95.5) | 0.87 | 85 (85.9) | 835 (96.4) | **<0.001** | 402 (95.0) | 464 (95.9) | 0.55 | 689 (96.5) | 162 (89.0) | 49 (98.0) | **<0.001** | 166 (96.0) | 149 (93.1) | 548 (96.1) | 0.25 | 149 (93.1) | 770 (95.8) | 0.15 |
|  | Positive | 30 (4.6) | 12 (6.8) |  | 2 (4.0) | 42 (4.5) |  | 14 (14.1) | 31 (3.6) |  | 21 (5.0) | 20 (4.1) |  | 25 (3.5) | 20 (11.0) | 1 (2.0) |  | 7 (4.0) | 11 (6.9) | 22 (3.9) |  | 11 (6.9) | 34 (4.2) |  |
